# C-952-08. Standardizing Frostbite Management in a Burn Center: Implementing Epoprostenol Monograph, Rewarming Guidelines, and Educational Tools

**DOI:** 10.1093/jbcr/irag033.124

**Published:** 2026-04-06

**Authors:** Anita Au, Shabana Lalji, Rimona Natanson, Benji Choo, Jasmine Segal, David Wallace, Ashley Callahan, Michelle Tong, Alice Shi

**Affiliations:** Sunnybrook Health Sciences Centre, Toronto, ON; Sunnybrook Health Sciences Centre, Toronto, ON; Sunnybrook Health Sciences Centre, Toronto, ON; Sunnybrook Health Sciences Centre, Toronto, ON; Sunnybrook Health Sciences Centre, Toronto, ON; Ross Tilley Burn Centre, Toronto, ON; Ross Tilley Burn Centre, Toronto, ON; Sunnybrook Health Sciences Centre, Toronto, ON; Sunnybrook Health Sciences Centre, Toronto, ON

## Abstract

**Introduction:**

Frostbite injuries, though infrequent, present unique challenges for burn centres due to their distinct pathophysiology and the absence of standardized treatment protocols. Inconsistent management can lead to delayed interventions, increased tissue loss, and poor patient outcomes. At our regional burn centre, a qualitative quality improvement initiative was undertaken to address these gaps through the development and implementation of a comprehensive, evidence-based frostbite management guideline.

**Methods:**

A multidisciplinary team designed a standardized frostbite protocol focused on three key components: (1) initiation of intravenous epoprostenol (a synthetic prostaglandin) for severe cases, (2) evidence-aligned rewarming procedures tailored to frostbite physiology, and (3) development of educational tools for both clinical staff and patients. Guideline development was informed by an extensive literature review (OVID Medline, 2020–2025), expert consensus, and a local needs assessment. A staff survey (83% response rate, n = 43/52) identified major gaps in frostbite care and highlighted the need for standardization and education. Implementation strategies included a structured physician order set, a bedside patient Kardex to facilitate interprofessional communication, and multi-modal education—didactic sessions, in-service trainings, and return demonstrations.

**Results:**

Following guideline implementation, preliminary outcomes demonstrated improved standardization and timeliness of care. In the majority of cases, epoprostenol therapy was initiated within 8 hours of patient admission, reflecting adherence to the new protocol. Staff feedback indicated enhanced confidence, improved clarity of treatment steps, and high usability of the supporting tools across clinical roles. Educational interventions were particularly valued for bridging knowledge gaps and improving interprofessional coordination.

**Conclusions:**

This initiative highlights the impact of a structured, evidence-informed approach to frostbite care within a burn center. By combining clinical guidelines with robust educational support, the project significantly improved consistency, timeliness, and confidence in frostbite management.

**Applicability of Research to Practice:**

The standardized guideline offers a replicable model for other burn centers seeking to optimize frostbite care. Ongoing monitoring will assess long-term clinical outcomes, adherence rates, and staff knowledge retention. Future efforts will focus on iterative refinement and dissemination of the guideline across broader regional and national networks.

**Funding for the Study:**

N/A.

